# Effects of sociodemographic characteristics and patients’ health beliefs on tuberculosis treatment adherence in Ethiopia: a structural equation modelling approach

**DOI:** 10.1186/s40249-017-0380-5

**Published:** 2017-12-15

**Authors:** Habteyes Hailu Tola, Mehrdad Karimi, Mir Saeed Yekaninejad

**Affiliations:** 1 Department of Epidemiology and Biostatics, School of Public Health, Tehran University of Medical Sciences, International Campus, Tehran, Iran; 2 Tuberculosis/HIV Research Directorate, Ethiopian Public Health Institute, P.O. Box 1242, /5654 Addis Ababa, Ethiopia

**Keywords:** Tuberculosis, Health belief, Treatment adherence, Structural equation modelling, Ethiopia

## Abstract

**Background:**

Patients’ beliefs are a major factor affecting tuberculosis (TB) treatment adherence. However, there has been little use of Health Belief Model (HBM) in determining the pathway effect of patients’ sociodemographic characteristics and beliefs on TB treatment adherence. Therefore, this study was aimed at determining the effect of sociodemographic characteristics and patients’ health beliefs on TB treatment adherence based on the HBM concept in Ethiopia.

**Methods:**

A cross-sectional study was conducted in Addis Ababa, Ethiopia among TB patients undertaking treatment. Thirty health centres were randomly selected and one hospital was purposely chosen. Six hundred and ninety-eight TB patients who had been on treatment for 1–2 month, were aged 18 years or above, and had the mental capability to provide consent were enrolled consecutively with non-probability sampling technique from the TB registration book until required sample size achieved. Structured questionnaires were used to collect data. Structural equation modelling was employed to assess the pathway relationship between sociodemographic characteristics, patients’ beliefs, and treatment adherence.

**Results:**

Of the 698 enrolled participants, 401 (57.4%) were male and 490 (70.2%) were aged 35 years and below. The mean age of participants was 32 (± 11.7) and the age range was 18–90 years. Perceived barrier/benefit was shown to be a significant direct negative effect on TB treatment adherence (*ß* = −0.124*, P* = 0.032). In addition, cue to action (*ß* = −0.68*, P* ≤ 0.001) and psychological distress (*ß = 0.08, P* < 0.001) were shown significant indirect effects on TB treatment adherence through perceived barrier/benefit.

**Conclusions:**

Interventions intended to decrease perceived barriers and maximize perceived benefits should be implemented to enhance TB treatment adherence. In addition, it is crucial that counselling is incorporated with the regular directly observed therapy program. Motivators (cue to actions) such as friends, family, healthcare workers, and the media could be used to promote TB treatment adherence.

**Electronic supplementary material:**

The online version of this article (10.1186/s40249-017-0380-5) contains supplementary material, which is available to authorized users.

## Multilingual abstracts

Please see Additional file 1 for translations of the abstract into the five official working languages of the United Nations.

## Background

Adherence is essential in the treatment of tuberculosis (TB) to achieve the required treatment success rate. However, due to long treatment duration, adherence to TB treatment is the most challenging factor affecting TB control.

Non-adherence can lead to poor treatment outcomes and drug resistance [1]. For example, evidence shows that the chance of developing multidrug-resistant TB (MDR-TB) among those who interrupt treatment for at least one day is higher than those who do not interrupt at all [2]. Besides the occurrence of drug resistance and treatment failure, non-adherence causes several health and socioeconomic related consequences [3–7], such as long hospitalization periods [3, 4, 6], delay in treatment completion [4], increased cost of treatment [4, 7], psychological morbidity [6–8], and increased mortality rate [5, 6].

Several factors have been found to contribute to TB treatment non-adherence [9–17]. Among them are: lack of knowledge about TB and its treatment [9, 10], disease-related stigma [11, 12], co-morbidity with other diseases [13–16], poverty [9, 11, 16], tobacco smoking and alcohol abuse [9, 14, 16], substance abuse [14], and drug side effects [10, 11]. In addition, disease severity [15, 17], psychological distress [18, 19], marital status and being on antiretroviral therapy [19], and forgetfulness [13] are also factors related to TB treatment non-adherence. A patient’s perception of his/her disease condition also plays a crucial role in treatment adherence [9, 17]. Patients who perceive less severity of the disease are less adherent than those who perceive high severity of the disease [9, 17]. These determinants of TB treatment adherence are interrelated and form a conceptual framework that is important for health behavior interpretation and prediction [20–22].

**Fig. 1:**
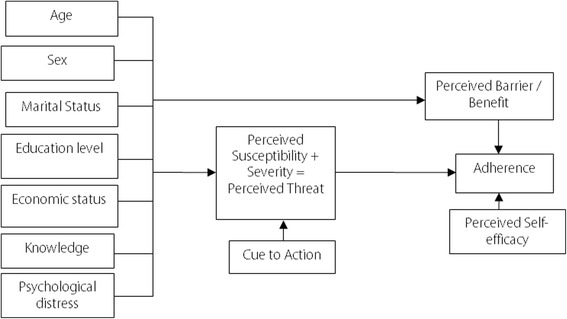
The HBM concept: the hypothesized model of the study

The health belief model (HBM) is among several social science conceptual frameworks that can predict and explain health beliefs among patients, including those related to treatment adherence [20–22]. However, the HBM has been criticized for being less applicable in the study of social and emotional components of behaviors [20]. According to the HBM conceptual framework (see Fig. 1), TB patients with specific sociodemographic characteristics adhere to their prescribed medication based on six belief-based circumstances [22, 23]. These are: 1) having minimal TB-related knowledge and motivation towards staying free of TB; 2) perceived susceptibility to TB and being convinced that TB is a severe medical problem; 3) belief that treatment adherence and TB medications are effective in curing TB; 4) belief that it is possible to obtain control over the barriers at acceptable psychological or tangible costs; 5) presence of internal or external stimuli, referred to as “cue to action,” that triggers health behavior of patients; and 6) self-efficacy belief to strictly follow treatment until the last dose. Accordingly, HBM can be an ideal framework to study the pathway relationship between sociodemographic characteristics and health beliefs that may influence TB treatment adherence.

Although several studies have reported the determinants of TB treatment adherence [10, 12, 13], few of them have used HBM as the guiding principle to determine factors associated with TB treatment adherence [19]. Moreover, pathway analysis is a useful statistical technique to determine the interrelation of sociodemographic characteristics and HBM domains [24–26]. However, there is no significant evidence showing the pathway relationship between sociodemographic factors and HBM domains relating to TB treatment adherence.

Ethiopia is among 22 high TB burden countries with 277/100000 prevalence cases recorded in 2011 [27]. The burden of treatment non-adherence is also relatively high (range: 10–21%) [10, 13, 19, 28]. However, information on how sociodemographic and HBM factors affect TB treatment adherence is limited.

Structural equation modelling (SEM) allows an objective method to evaluate the adequacy of the theoretical model to the observed data. Use of latent variables in SEM permits estimation of relationships between theoretically interrelated constructs that are free from the effects of measurement. The approach supports the development and testing of models, as well as the construction of alternative models concerning their relative fit to the collected data. In general, SEM allows the testing of important theories to be developed within a substantive domain. Thus, to understand the direct and indirect impacts of TB disease knowledge, psychological distress, sociodemographic variables, and the six HBM domains on TB treatment adherence, the SEM approach is more effective than an ordinary regression model.

This study was aimed at determining the direct and indirect effects of sociodemographic characteristics and patients’ health beliefs on TB treatment adherence in Ethiopia, based on the HBM conceptual framework using SEM.

## Methods

### Study area and population

This study was conducted in Addis Ababa, Ethiopia. Patients diagnosed with active TB were enrolled consecutively by non-probability sampling technique using TB registration number from 30 health centers (HCs) and one hospital. The 2014 population stabilization census report estimated the population of Addis Ababa at three million [29].

In 2015, there were 200 000 new TB cases in Ethiopia, which ranked the country 10th among the 22 high TB burden countries in the world and 4th in Sub-Saharan Africa, following Nigeria, South Africa, and the Democratic Republic of Congo [30].

Every year, TB kills an estimated 30 000 people in the country [30]. Although an estimated national TB treatment success rate was 89% in 2013 [31], recent studies conducted in different parts of the country indicate that a considerable proportion of patients had poor treatment outcomes [32, 33]. For instance, a study in southern Ethiopia revealed that unsuccessful treatment rate was 56.7% [32], while a study in the northern part of the country indicated a 34.7% unsuccessful outcome rate [33]. In Ethiopia, all health facilities treat TB cases using directly observed treatment (DOT) [34].

### Study design and sampling

A cross-sectional study was conducted among TB patients who were on DOT in Addis Ababa. Addis Ababa has 10 sub-city administrations with 53 HCs, 10 public hospitals, and several private healthcare institutions.

Out of all HCs in Addis Ababa, 30 were randomly selected as study sites. In addition, one referral hospital was purposely selected as it is the only public hospital in the city that is dedicated to treat both MDR-TB and drug-susceptible TB patients.

The main inclusion criteria were: TB patients, who had been on treatment for 1–2 months, were aged 18 years or above, and were mentally capable to provide consent. The reason behind enrolling TB patients who had been on treatment for 1–2 months was to measure their psychological distress in the past month, as recommended by Kessler et al. [35], and to measure the treatment adherence level in the past month using the visual analogue scale (VAS) [36]. In addition, the 2 month maximum limit was to include sufficient participants to achieve the required sample size within specified period.

The non-adherence level in TB patients under normal DOT was 20%, as reported previously [28]. This was used as a population proportion to determine the sample size. Moreover, 5% type I error, 1.5 design effect, 80% power, and 10% contingency sample were considered for total sample size estimation.

It was calculated that 684 participants had to be included in the study. However, 698 TB patients who fulfil the inclusion criteria were enrolled in the study consecutively with non-probability sampling technique from the TB registration book using the patients’ identification numbers. More participants were enrolled in order to increase estimation power and to minimize the chance of selection bias. Selection bias might be introduced due to the consecutive (sequential) non-probability sampling technique used through missing important participants at the end of registry that may differ from those enrolled at the beginning of registry.

### Data collection

Demographic data were collected using a structured questionnaire. Economic status was assessed using 10 questions centred on the ownership of basic assets. The responses to these questions were recorded by yes = 1 and no = 0. The composite index of the economic score was computed using one dimension categorical principal component analysis (CPCA).

To assess TB disease and treatment knowledge, structured questions were also employed. The response to each question was recorded using a three-point Likert scale (correct = higher score, incorrect and don’t know = lower score), and the total knowledge level was scored using one dimension CPCA.

Similarly, to collect information on the six HBM domains, structured questions were used and their responses were recorded using a five-point Likert scale (ranging from “strongly disagree” = lowest score and “strongly agree” = highest score). The total composite index of each HBM domain was scored using one dimension CPCA.

Perceived threat was a variable composed of the sum of perceived susceptibility and perceived severity after computing the separate composite index using one dimension CPCA. Similarly, perceived barrier/benefit was determined by subtracting the total score of perceived benefit from the total score of perceived barrier. The presence of psychological distress in the last month was assessed using Kessler et al.’s 10-item scale [35] and the response to each item was recorded using a five-point Likert scale (ranging from “every time” = highest score to “none of the time” = lowest score).

The total composite index of psychological distress was scored using the total sum method, as recommended by the Kessler et al.’s [35]. Questionnaires used for data collection were validated before actual data collection by a pilot study at selected study sites, and all tools were found to be valid and reliable at the recommended Cronbach’s alpha for ordinal scales and Kuder-Richardson Formula 20 for binary questions above 0.7.

The VAS was used to assess patients’ treatment adherence. Although VAS is not the gold standard for measuring treatment adherence, it is an important tool for screening patients’ adherence in resource-limited settings and it is relatively non-influenced by response bias [36].

Patients estimated their own adherence using VAS, after it was carefully explained to them. The VAS scores ranged from 0% (not a single dose taken) to 100% (not a single dose missed) to the question: “How many of your scheduled medications did you take in the last 30 days (percentage)?” Participants who estimated their adherence level to be above 90% were considered as adherent based on the World Health Organization (WHO) adherence definition [37] and national TB-HIV and leprosy treatment guidelines [38]. Moreover, patients who had been on treatment for at least one month and interrupted the treatment for two months or more consecutively were considered as lost to follow-up [38]. Thus, the TB patients belonged to one of three categories: (1) those who interrupted their treatment and guessed their adherence level as being less than 90% on VAS, (2) those who interrupted their treatment for more than 10 doses due to experiencing adverse effects, and (3) those who were lost to follow-up were considered non-adherent. However, patients who were interrupted their treatment less than 10% and guessed their adherence level as being above 90% on VAS were considered as adherent. Participants who interrupted treatment due to side effects or were lost to follow-up were interviewed at their homes after agreeing to this via a telephone call.

### Data analysis

Data were checked for errors and statistical assumptions were assessed before the main data analysis. Frequency (percentage) distribution for categorical variables was reported as descriptive statistics. In the primary analysis, the chi-square test was used to compare the distribution of categorical variables between adherence statuses. The hypothesized structural model that shows the interrelationship between variables was drawn graphically (see Fig. 1). In the path analysis as SEM, all parameters were estimated using maximum likelihood method, and level of significance was checked by bias corrected percentile method as a bootstrapping.

The direct impact of participants’ characteristics such as age, sex, education, marital status, economic status, TB knowledge, psychological distress, and cue to action, and perceived threat (the sum of perceived susceptibility and perceived severity) and perceived barrier/benefit (perceived barrier minus perceived benefit) were assessed. In addition, the direct effects of perceived threat and perceived barrier/benefit on adherence were estimated. The primary path model was modified several times by introducing correlations between variables to improve the goodness of fit indices. The validity of model fitness was assessed using the chi-square statistic and various important fit indices such as the standardized root mean squared residual (SRMR), root mean squared error of approximation (RMSEA), comparative fit index (CFI), Tucker-Lewis index (TLI), and goodness of fit index (GFI). The IMB, Statistical Package for the Social Sciences (SPSS) and Analysis of Moment Structure (AMOS) added to SPSS version 20, Chicago were used for data analysis.

## Results

### Participants’ sociodemographic characteristics

Four hundred-one (57.4%) male and 297 (42.6%) female TB patients were enrolled in this study. The mean age of participants was 32 (*SD* ± 11.7) years and the age range was 18–90 years. The majority (70.2%) of participants were aged below 35 years and 474 (67.9%) were not married. More than half (55.4%) were likely to have had psychological distress, at the cut-off point of 16 (out of a maximum score of 50). The mean score of knowledge was 30.5 (± 4.7) and the mean score of perceived susceptibility was 28.2 (± 6.6). The mean scores of perceived severity, perceived barrier, perceived benefit, cue to action, and perceived self-efficacy were (36.9 ± 5.0), (65.5 ± 16.6) (32.8 ± 4.1), (31.7 ± 5.3), and (51.5 ± 5.6), respectively. The overall treatment adherence level in past month (prior to interview date) was 80.5% (see Table 1).

**Table 1:** Participants’ sociodemographic characteristics (*n* = 698)

Variable		*n* (%)	95% CI* for %
Sex	Female	297 (42.6)	38.9–46.3
	Male	401 (57.4)	53.8–61.1
Age group (years)	35 and below	490 (70.2)	66.7–73.5
	Above 35	207 (29.7)	26.4–33.2
Education level	Illiterate	115 (16.5)	13.9–19.4
	Elementary	275 (39.4)	35.8–43.1
	High school	224 (32.1)	28.7–35.7
	College diploma and above	84 (12.0)	9.8–14.7
Marital status	Married	224 (32.1)	28.7–35.7
	Unmarried	474 (67.9)	64.4–71.3
Economic status	Low	317 (45.4)	41.8–49.1
	High	381 (54.6)	50.9–58.2
Distress	Likely to not be distressed	311 (44.1)	40.9–48.3
	Likely to have symptom(s) of distress	387 (55.4)	51.7–59.1
Adherence	Adherent	562 (80.5)	77.4–83.3
	Non-adherent	136 (19.5)	16.7–22.6

*confidence interval

Participants in the low economic score were more likely to be non-adherent (26.5%) than those in the high economic score category (13.6%) (*P <* 0.001). Those who had high school education or lower (20.7%) were more likely to be non-adherent than those with a college diploma or above (10.7%) (*P =* 0.030). Although the difference was not statistically significant (*P =* 0.456), unmarried participants (20.3%) were slightly more likely to be non-adherent than married (17.9%) participants (see Table 2).

**Table 2:** Participants’ sociodemographic characteristics and TB treatment adherence (*n* = 698)

Variable		Adherent *n* (%)	Non-adherent *n* (%)	*P* -value
Age group (years)	35 and below	392 (80.0)	98 (20.0)	
	Above 35	169 (81.6)	38 (18.4)	0.617
Sex	Female	234 (78.8)	63 (21.2)	
	Male	328 (81.8)	73 (18.2)	0.321
Marital status	Married	184 (82.1)	40 (17.9)	0.456
	Unmarried	378 (79.7)	96 (20.3)	
Education level	High school and below	487 (79.3)	127 (20.7)	
	Diploma and above	75 (89.3)	9 (10.7)	0.030
Economic status	Low	233 (73.5)	84 (26.5)	
	High	329 (86.4)	52 (13.6)	<0.001
Psychological distress	Likely to not be distressed	252 (81.0)	59 (19.0)	
	Likely to have symptom(s)	310 (80.1)	77 (19.9)	< 0.001

### Pathway analysis

**Fig. 2:**
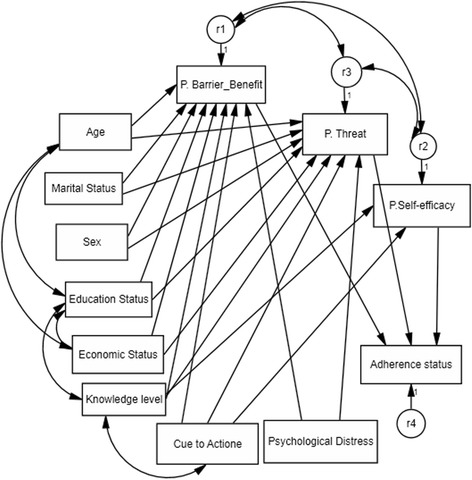
Modified path analysis model with the added associations between variables (r1, r2, and r3 show residual variance for dependent variables)

Our pathway analysis model contained seven sociodemographic characteristics as exogenous observed variables: age, sex, education level, marital status, economic status, knowledge level, and psychological distress. In addition, the model contained perceived threat and perceived barrier/benefit as dependent variables for the participants’ sociodemographic characteristics, and adherence status as the main outcome variable for perceived threat and perceived barrier/benefit. Perceived self-efficacy and cue to action were also portrayed in this model as exogenous variables (independent variables) through perceived threat and perceived barrier/benefit (see Figs. 1 and 2).

Table 3 depicts the fitness indices of conceptual and modified models. The modified model, in which an association was added between exogenous independent variables, showed acceptable goodness of fit to the data with SRMR (0.047), RMSEA (0.058), CFI (0.96), GFI (0.98), and TLI (0.93). The additional associations that were added to improve the fit indices in the modified model were based on correlations between knowledge and cue to action (0.226), age and education (−0.228), perceived barrier/benefit and perceived threat (−0.375), perceived self-efficacy and perceived threat (0.341), and perceived barrier/benefit and perceived self-efficacy (−0.510). All correlations were significant at *P* < 0.001.

**Table 3:** Model fitness indices for modified and conceptual models

Indices	*χ* ^2^	DF	*χ* ^2^/DF	SRMR	RMSEA	CFI	GFI	TLI
Conceptual model	765.69	45	17.05	0.10	0.15	0.62	0.85	0.44
Modified model	114.78	34	3.38	0.047	0.058	0.96	0.98	0.93
Acceptable threshold			< 4	< 0.1	< 0.08	> 0.90	> 0.90	> 0.90

*χ* ^2^ *:* chi-square, DF: degree of freedom

**Fig. 3:**
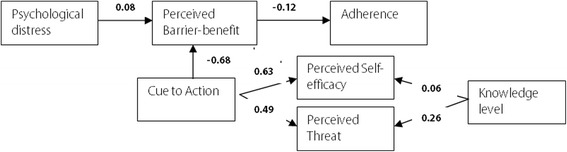
Variables with significant standardized direct and total effects

Table 4 shows the standardized direct, indirect, and total effects of each exogenous variable on intermediate dependent variables and on the main outcome variable. In addition, Fig. 3 shows variables that revealed significant direct and total effects.

**Table 4:** Standardized direct, indirect, and total effects of sociodemographic variables in the model on TB treatment adherence

		Standardized effect		
Dependent variables	Predictor variables	Direct	Indirect	Total
Adherence	Age	–	0.006	0.006
	Sex	–	0.001	0.001
	Economic status	–	−0.003	−0.003
	Education level	–	0.001	0.001
	Marital status	–	−0.004	0.004
	Knowledge level		0.018	0.018
	Psychological distress		−0.005	−0.005
	Cue to action	–	0.067*	0.067*
	Perceived barrier/ benefit	−0.124*	–	−0.124*
	Perceived self-efficacy	−0.075	–	−0.075
	Perceived threat	0.061	–	0.061
Perceived barrier/benefit	Age	−0.037		−0.037
	Sex	−0.007		−0.007
	Economic status	0.020		0.020
	Education level	−0.010		−0.010
	Marital status	−0.038		−0.038
	Knowledge level	−0.049		−0.049
	Psychological distress	0.076*		0.076*
	Cue to action	−0.677*		−0.677*
Perceived threat	Age	0.028	–	0.028
	Sex	0.005	–	0.005
	Economic status	−0.015	–	−0.015
	Education level	−0.025	–	−0.025
	Marital status	0.015	–	0.015
	Knowledge level	0.262*	–	0.262*
	Psychological distress	0.068	–	0.068
	Cue to action	0.495*	–	0.495*
Perceived self-efficacy	Sex	–	–	–
	Knowledge level	0.059*	–	0.059*
	Psychological distress	–	–	–
	Cue to action	0.628*	–	0.628*

*effect significance at *P* < 0.05

Perceived barrier/benefit (*ß* = −0.124*, P* = 0.032) was shown to be a significant direct and total negative effect on treatment adherence, while cue to action was shown to be a significant positive indirect and total effect (*ß* = 0.07*, P <* 0.001). However, perceived threat did not have either a direct or indirect significant effect on adherence (see Table 4). Cue to action was shown to be a significant direct and total negative effect (*ß =* −0.68*, P <* 0.001) on perceived barrier/benefit, while psychological distress was shown to be a significant direct and total positive effect (*ß* = 0.08*, P* = 0.002) on perceived barrier/benefit (see Table 4 and Fig. 3). In addition, cue to action (*ß* = 0.50*, P* < 0.001) and knowledge (*ß* = 0.26*, P* < 0.001) were shown to be significant direct and total positive effects on perceived threat (see Table 4). Similarly, cue to action (*ß* = 0.63*, P* < 0.001) and knowledge (*ß* = 0.06*, P* < 0.049) were shown to be significant direct and total positive effects on perceived self-efficacy. However, none of other variables were shown to be significant effects on either perceived barrier/benefit or perceived threat (see Table 4 and Fig. 3].

## Discussion

Adherence to prescribed medication is vital in treating diseases. According to the WHO recommendation, a TB patient must take at least 90% of prescribed medication to be cured or achieve completion of treatment [37]. However, a considerable proportion of TB patients are failing to adhere to the prescribed medication to the end of their follow-up period [13, 15, 16, 19, 28].

Adherence to long-term treatment is a complex phenomenon, influenced by wide range of interrelated factors [39]. These factors form a conceptual framework by interacting with each other and puts pressure on the patient’s tolerance ability to follow the treatment strictly [22, 23]. This means, factors that associated with TB treatment adherence influence each other and finally affect the outcome behaviour directly or indirectly in conceptual manner. Therefore, this study was aimed at determining the effects of sociodemographic variables and patients’ health beliefs on TB treatment adherence based on the HBM concept.

In this study, perceived barrier/benefit was shown to be a significant direct negative effect on TB treatment adherence, while cue to action was shown to be a significant indirect positive effect through perceived barrier/benefit. In addition, cue to action and psychological distress were shown to be significant direct effects on perceived barrier/benefit. Moreover, TB disease knowledge and cue to action were shown to be significant direct effects on perceived threat and perceived self-efficacy.

As previously reported, TB patients must believe in the benefit of treatment and TB medications, and that it is possible to obtain control over barriers at acceptable psychological or tangible costs [22, 23]. According to the expectancy-value theory, individuals rationally choose non-adherence when perceived barriers or costs of treatment outweigh the expected perceived benefits [40]. Although we could not find any study that used the HBM to determine factors affecting TB treatment adherence, one systematic review conducted on general medication adherence predictors using psychological models highlighted that perceived barriers were among the factors that predicted medication adherence [41]. Similarly, our study indicated that perceived barrier/benefit negatively affects TB treatment adherence, and that cue to action affects treatment adherence indirectly through perceived barrier/benefit. This means, if motivation (cue to action) from media, healthcare workers, family members, friends etc. is low, then the patient tends to be non-adherent through exaggerating the normal level of barrier/benefit. That is patients who perceive high barrier/benefit are more likely to be non-adherent if there is low motivation from different sources. This finding is consistent with a previous study that found peer and family support, and attractive healthcare worker-related behaviors etc. increased TB treatment adherence through increasing a patient’s motivation [42].

Psychological distress was shown to be a significant direct effect on perceived barrier/benefit, which directly affects treatment adherence. This indicates that patients with high psychological distress have more chance to be non-adherent through increased perceived barrier/benefit score. In another way patients who have high psychological distress symptom(s) are more likely perceive high barrier/benefit and finally enter to non-adherence. Although we could not find literature reporting the indirect effect of psychological distress on treatment adherence through perceived barrier/benefit, it has been reported to have a strong significant direct effect on TB treatment adherence [8, 18, 19].

Perceived threat was directly predicted by TB disease and its treatment knowledge. A high knowledge score leads to awareness of the seriousness of TB disease. That is patients who have more knowledge about the seriousness of TB disease are more likely to be adherent than those who don’t have sufficient knowledge. However, perceived threat did not result in significant prediction of TB treatment adherence. A previous study indicated that perceived high seriousness of a disease was strongly associated with treatment adherence [17]. This finding contradicts our finding. However, lack of association between perceived threat and adherence is quite consistent with usual conclusion in health education and health behaviour change concepts. Similarly, cue to action was shown to be indirect significant effect on treatment adherence through perceived threat. However, perceived threat was not shown a significant direct effect on treatment adherence. Although we could not find similar studies showing the effect of cue to action on perceived threat, one systematic review indicated that peers, family, healthcare workers, media etc. were associated with a patient’s motivation for treatment adherence [42].

Although several studies revealed that perceived self-efficacy has a high effect on medication adherence [41, 43], it did not show a significant effect on TB treatment adherence in this study. This is probably due to the difference in the study populations, instruments used for data collection, and analysis methods. However, cue to action and knowledge were positively associated with perceived self-efficacy.

This study’s questionnaire was administered by health professionals working at the selected HCs. Health professionals might have over-reported the adherence level due to fear that supervisors may question them over poor performance. On the other hand, participants themselves might have underreported the barriers related to healthcare facilities and healthcare workers, as the interviewers themselves were health workers. Thus, these might be sources of bias for overestimation and underestimation of the adherence level. In addition, although various social, economical, health system, and individual behavioural-related factors affect TB treatment adherence, we selected a few sociodemographic factors and health beliefs due to the limitation of the HBM theoretical concept to include all factors influencing TB treatment adherence. Thus, considering other theoretical models that can accommodate a wide range of factors influencing TB treatment adherence will be crucial in the future. Despite these limitations, we believe that the results of this study would be less likely to be biased.

## Conclusions

Perceived barrier/benefit was shown to be a significant direct negative effect on TB treatment adherence. Adherence level was indirectly affected by cue to action and psychological distress through perceived barrier/benefit. Therefore, interventions intended to reduce perceived barriers and maximize perceived benefits should be implemented to improve treatment adherence. In addition, involving patients’ family and friends, the media, and healthcare workers as motivators (cue to action) in promoting treatment adherence is vital. Incorporating psychological counselling with regular DOT could also enhance TB treatment adherence.

## Additional files

Additional file 1: Multilingual abstracts in the five official working languages of the United Nations. (PDF 422 kb)

## Abbreviations

CFI: Comparative fit index
CPCA: Categorical principal component analysis
DOT: Directly observed treatment
GFI: Goodness of fit index
HBM: Health belief model
HC: Health center
MDR-TB: Multidrug-resistant TB
RMSEA: Root mean squared error of approximation
SEM: Structural equation modeling
SRMR: Standardized root mean squared residual
TB: Tuberculosis
TLI: Tucker-Lewis index
VAS: Visual analogue scale
WHO: World Health Organization
