# Supplementary material for: Effects of sociodemographic characteristics and patients’ health beliefs on tuberculosis treatment adherence in Ethiopia: a structural equation modelling approach
Source: Infect Dis Poverty. 2017 Dec 15;6:167. doi: 10.1186/s40249-017-0380-5 (PMC5731079; doi:10.1186/s40249-017-0380-5)
Supplement: Supplementary file 1 — Multilingual abstracts in the five official working languages of the United Nations. (PDF 422 kb) [file 40249_2017_380_MOESM1_ESM.pdf]

Translation of the abstract into the five official working languages of the United Nations

آثار الخصائص الاجتماعية والديموغرافية والمعتقدات الصحية للمرضى على التقيد بالعلاج المقدم لمرض السل في إثيوبيا: نهج تصميم معادلة هيكلية.

هابتيس هايلو تولا، ومهرداد كاريمي، ومير سعيد يكانينجاد.

ملخص
مقدمة: <bx/> تعتبر معتقدات المرضى عاملاً رئيسياً يؤثر على التقيد بعلاج الدرن مرض السل. رغم ذلك، استخدم نموذج المعتقدات الصحية على نطاق ضيق في تحديد أثر الخصائص والمعتقدات الاجتماعية والديموغرافية للمرضى والمعتقدات على الالتزام بعلاج السل. وعليه، هدفت هذه الدراسة إلى تحديد تأثير الخصائص الاجتماعية والديموغرافية والمعتقدات الصحية للمرضى على الالتزام بعلاج السل بناءً على مفهوم نموذج المعتقدات الصحية في إثيوبيا.
طرق البحث: <bx/> أجريت دراسة مقطعية في أديس أبابا، إثيوبيا على مرضى السل الذين يتلقون العلاج. جرى اختيار ثلاثين مركز صحي ومستشفى واحد عشوائياً لأغراض إجراء هذه الدراسة. جرى إشراك ستمئة وثمانية وتسعون مريضاً بالسل خضعوا للعلاج لمدة تراوحت من شهر إلى شهرين، وكانوا يبلغوا ثمانيةً عشر عاماً أو أكثر من العمر، ممن يتمتعون بالقدرة العقلية على الموافقة في الدراسة باستخدام تقنية أخذ عينات لا تقبل احتمال من سجل مرضى السل حتى تم الوصول إلى حجم العينة. واستخدمت استبيانات منظمة لجمع البيانات. واستخدمت تصميم معادلة هيكلية لتقييم مسار العلاقة بين الخصائص الاجتماعية والديموغرافية ومعتقدات المرضى، والالتزام بعلاج مرض السل.
النتائج: <bx/> بلغ العدد الإجمالي للمشاركين في الدراسة 698 مريضاً، 401 (أي ما نسبته 57.4%) كانوا من الذكور و 490 مريضاً (أي ما نسبته 70.2%) كانت أعمارهم خمسة وثلاثين عاماً أو أقل. وكان متوسط أعمار المشاركين في الدراسة اثنين وثلاثين عاماً (± 11.7) والفئة العمرية من ثمانية عشرة عاماً إلى تسعين عاماً. واتضح بأنَّ الحاجز/الفائدة المتصور/المتصورة كان لها تأثير سلبي مباشر كبير على الالتزام بعلاج مرض السل (*ß=-0.124, P= 0.032*). بالإضافة إلى ذلك، بدا بأنَّ إشارة بدء العمل (*ß =-0.68*، ف ≤<ex/> *0.001*) والضغط النفسي (*ß = 0.08*، ف *< 0.001*) كان لها تأثيرات غير مباشرة كبيرة على الالتزام بعلاج مرض السل من خلال الحاجز والفوائد المتصورة.
الاستنتاجات: <bx/> ينبغي تنفيذ التدخلات الرامية إلى تقليل الحواجز المتصورة، وتحقيق أكبر عدد من الفوائد لزيادة نسبة الالتزام بعلاج مرض السل. وبالإضافة إلى ذلك، من الأهمية بمكان دمج تقديم المشورة مع برنامج العلاجي الذي يخضع للمراقبة المباشرة. ويمكن استخدام المحفزات (إشارة بدء العمل)، مثل الأصدقاء، والأسرة، والعاملين في مجال الرعاية الصحية، ووسائل الإعلام لتطوير نسبة الالتزام بعلاج مرض السل.

Translated from English version into Arabic by Sofyan barhomeh, through

TRANSLATORS
WITHOUT BORDERS

埃塞俄比亚结核病患者的社会人口学特征及其健康信念对结核病治疗依从性的影响：一项结构方程建模研究

Habteyes Hailu Tola, Mehrdad Karimi and Mir Saeed Yekaninejad

摘要:
引言：患者的健康信念是影响结核病（TB）治疗依从性的主要因素之一。然而，健康信念模型（HBM）很少被用于研究患者的社会人口学特征和信念对结核病治疗依从性的影响。因此，本研究旨在根据埃塞俄比亚的 HBM 理念，明确社会人口学特征和患者健康信念对结核

病治疗依从性的影响。
方法：在埃塞俄比亚亚的斯亚贝巴对接受治疗的结核病患者进行横断面研究。随机抽取 30 家健康中心及特意选取的 1 家医院。通过连续非概率抽样方法从 TB 患者中抽取满足样本量的 689 例已治疗 1～2 月的患者，年龄为 18 岁及以上且精神状态正常。采用结构化调查问卷收集数据。通过结构方程建模评估社会人口学特征和患者信念与治疗依从性之间的联系。
结果：698 例参与者中，401 例（57.4％）为男性，490 例（70.2％）的年龄为 35 岁及以下。平均年龄为 32 岁（±11.7），年龄范围为 18～90 岁。知觉障碍或知觉利益对 TB 治疗依从性有显著的直接负面影响（ß= -0.124，*P* = 0.032）。 另外，行动（ß= -0.68，*P*≤0.001）和心理困扰（ß= 0.08，*P* <0.001）通过知觉障碍或知觉利益对结核病治疗依从性有显著的间接影响。
结论：应实施旨在减少知觉障碍的干预措施，并最大限度地提高知觉利益，以提高结核病治疗依从性。另外，咨询与常规的直接观察治疗方案相结合是至关重要的。可采取激励因素(行动提示)，如朋友、家庭、医护人员和媒体可以提高结核病治疗依从性。

Translated from English version into Chinese by Peng Song, through

CHINESE CENTER FOR DISEASE CONTROL AND PREVENTION
NATIONAL INSTITUTE OF PARASITIC DISEASES

Effets des caractéristiques sociodémographiques et des croyances des patients en matière de santé sur l'adhésion au traitement contre la tuberculose en Ethiopie : une approche basée sur les modèles d'équations structurelles.

Habteyes hamadou Tola, Mehrdad Karimi et Mir Saeed Yekaninejad

Résumé
Introduction : les croyances des patients sont un facteur déterminant altérant l'adhésion au traitement contre la tuberculose (TB). Pourtant, le Health Belief Model (HBM) n'a été que très peu utilisé pour déterminer l'incidence des caractéristiques sociodémographiques des patients et de leurs croyances sur leur adhésion au traitement contre la tuberculose. Ainsi, l'objectif de cette étude était de déterminer les effets des caractéristiques socio-démographiques et des croyances des patients en matière de santé sur l'adhésion au traitement antituberculeux en Ethiopie, en se basant sur le modèle HBM.
Méthode : une étude transversale menée à Addis Ababa, en Ethiopie, auprès de patients sous traitement antituberculeux. Trente centres de santé ont été choisis de façon aléatoire, et un hôpital a été choisi intentionnellement. Six cent quatre-vingt-dix-huit patients atteints de tuberculose, sous traitement depuis un à deux mois, âgés de 18 ans minimum et ayant la capacité mentale de donner leur consentement, ont été inscrits successivement selon la technique d'échantillonnage non-aléatoire, à partir du livre de registre des patients atteints de tuberculose, jusqu'à ce que la taille d'échantillons requise soit atteinte. Des questionnaires structurés ont été utilisés afin de recueillir les données. Le modèle des équations structurelles a été utilisé pour évaluer la corrélation entre les caractéristiques sociodémographiques, les croyances des patients et l'observance du traitement.
Résultats : parmi les 698 participants inscrits, 401 (57,4%) étaient des hommes, et 490 (70,2%) étaient âgés de 35 ans ou moins. L'âge moyen des participants était de 32 ans (± 11,7) et la tranche d'âge de 18 à 90 ans. La perception des obstacles et des avantages s'est révélée avoir un effet négatif influençant directement l'adhésion au traitement antituberculeux (*ß* = -0.124, *P* = 0,032). De plus, certains indices à l'action (*ß* = -0.68, *P* ≤ 0.001) et un état de détresse psychologique (*ß = 0.08, P* < 0.001) ont montré qu'ils pouvaient avoir des effets indirects significatifs sur l'adhésion au traitement

antituberculeux, liés aux obstacles et avantages perçus.
Conclusion : des interventions visant à réduire la perception des obstacles et maximiser celle des avantages doivent être mises en œuvre afin d'améliorer l'adhésion au traitement antituberculeux. De plus, il est crucial qu'un suivi psychologique soit intégré au programme de thérapie standard directement observé. Les facteurs de motivation (indices à l'action, ou déclencheurs) tels que les amis, la famille, le personnel de santé et les médias pourraient être utilisés pour promouvoir l'adhésion au traitement antituberculeux.

Translated from English version into French by charlotteman, through

Влияние социально-демографических характеристик и убеждений пациентов в отношении здоровья на приверженность к лечению от туберкулеза в Эфиопии: метод моделирования структурного уравнения

Хабтейес Хайлу Тола, Мехрдад Карими и Мир Саид Еканиньяд

Аннотация

Введение: Убеждения пациентов являются основным фактором, влияющим на приверженность к лечению от туберкулеза (ТБ). При этом Модель убеждений в отношении здоровья (МУЗ) практически не использовалась для изучения влияния социально-демографических характеристик и убеждений на приверженность пациентов к лечению от ТБ . Поэтому это исследование, проведенное в Эфиопии, было направлено на изучение влияния социально-демографических характеристик и убеждений пациентов в отношении здоровья на приверженность к лечению от ТБ на основе концепции МУЗ.
Методы: Было проведено одномоментное исследование среди больных ТБ, получающих лечение, в Аддис-Абебе, Эфиопия. Было выбрано тридцать медицинских учреждений случайным образом и одна больница целенаправленно. Сто девяносто восемь больных туберкулезом, которые находились на лечении 1 – 2 месяца, в возрасте 18 лет и старше, обладающих ментальной способностью для предоставления согласия, были зачислены в последовательном порядке с помощью не-вероятностного отбора из базы регистрации случаев ТБ до достижения необходимого размера выборки. Для сбора данных были использованы структурированные вопросники. Для оценки взаимосвязи между социально-демографическими характеристиками, убеждениями пациентов и приверженностью к лечению использовали метод моделирования структурированного уравнения.
Результаты: Из 698 включенных участников исследования 401 пациент (57,4%) был мужского пола и 490 пациентов (70,2%) были в возрасте 35 лет и менее. Средний возраст участников составил 32 (± 11,7) года и диапазон возраста – от 18 до 90 лет. Было показано, что соотношение «осознаваемое препятствие/осознаваемая польза» оказывает значительное прямое негативное влияние на приверженность к лечению от ТБ (*ß* =-0,124, $P = 0{,}032$). Кроме того, стимул к действию (*ß* =-0,68, $P \leq 0{,}001$) и психологический дистресс (*ß = 0,08*, $P < 0{,}001$)

в значительной степени оказывали косвенное влияние на приверженность к лечению от туберкулеза в зависимости от соотношения «осознаваемое препятствие/осознаваемая польза».
Выводы: Для повышения приверженности к лечению от ТБ должны предприниматься действия, направленные на снижение осознаваемых препятствий и максимальное понимание пользы от собственных действий. Кроме того, крайне важно, чтобы консультирование было включено в стандартную программу лечения под непосредственным наблюдением. Для поощрения приверженности к лечению от ТБ могут использоваться мотиваторы (те, кто стимулирует к действию), такие как друзья, семья, медицинские работники и средства массовой информации.

Translated from English version into Russian by Leila Usmanova, through

TRANSLATORS
WITHOUT BORDERS

Efectos de las características sociodemográficas y las creencias de salud de los pacientes en el cumplimiento del tratamiento de la tuberculosis en Etiopía: un enfoque a partir del modelo de ecuaciones estructurales

Habteyes Hailu Tola, Mehrdad Karimi y Mir Saeed Yekaninejad

Resumen

Introducción: Las creencias de los pacientes son un factor clave en el cumplimiento del tratamiento de la tuberculosis. Sin embargo, se ha hecho poco uso del Modelo de Creencias de Salud (MCS) para determinar el efecto del desempeño de las creencias y las características sociodemográficas del paciente en el cumplimiento del tratamiento de la tuberculosis. Por lo tanto, este estudio tuvo como finalidad determinar el efecto de las características sociodemográficas y las creencias de salud de los pacientes en el cumplimiento del tratamiento de la tuberculosis según el concepto del MCS en Etiopía.
Métodos: Se realizó un estudio transversal en Adís Abeba, Etiopía entre pacientes con tuberculosis que estuviesen en tratamiento. Se seleccionaron treinta centros de salud de manera aleatoria y se eligió deliberadamente un hospital. Se incluyeron de manera consecutiva y hasta alcanzar el tamaño de muestra requerido, seiscientos noventa y ocho pacientes del libro de registro de tuberculosis que habían recibido tratamiento durante 1 a 2 meses, de la edad de 18 años o más y con la facultad mental de dar su consentimiento. Se utilizó la técnica de muestreo no probabilístico, . Se utilizaron cuestionarios estructurados para recoger datos. Se utilizó el modelo de ecuaciones estructurales para evaluar la relación desempeño entre las características sociodemográficas, las creencias de los pacientes y el cumplimiento del tratamiento.
Resultados: De los 698 participantes inscritos, 401 (57.4%) eran hombres y 490 (70.2%) tenían 35 años o menos. La edad media de los participantes fue de 32 (± 11,7) y el rango de edad fue de 18 a 90 años. Se demostró que los costos de acción y los beneficios percibidos poseen un efecto negativo importante en el cumplimiento del tratamiento de la tuberculosis ($ß$ = -0.124, P = 0.032). Además,

se demostró que las señales de acción (*ß* = -0.68, $P \leq 0.001$) y el trauma psicológico (*ß = 0.08*, $P < 0.001$) tienen un efecto indirecto significativo en el cumplimiento del tratamiento de la tuberculosis mediante los costos y beneficios percibidos.

Conclusiones: Se deben implementar intervenciones con el fin de disminuir los costos de acción o barreras percibidos y aumentar los beneficios percibidos para mejorar el cumplimiento del tratamiento de la tuberculosis. Además, es fundamental que el programa regular de terapia directamente observada cuente son servicios de asesoría incorporados. Se podrían utilizar factores motivacionales (señales de acción) tales como amigos, familiares, personal de la salud y los medios de comunicación para promover el cumplimiento del tratamiento de la tuberculosis.

Translated from English version into Spanish by María Jesús González, through

TRANSLATORS
WITHOUT BORDERS
